# Chicago Pathological Society

**Published:** 1888-08

**Authors:** 


### CHICAGO PATHOLOGICAL SOCIETY.

(Stenographically reported for the JOURNAL.)

STATED MEETING, July 9.

President Professor I. N. Danforth in the chair.

The President reported a case of "Tubercular Disease of the Kidneys and Bladder," and presented the diseased organs.

He said that the patient was a young man 30 years of age. About four years prior to his death he began to experience pain in the region of the left kidney, and shortly after that the urine became moderately purulent and there was a moderate amount of albumen in the urine. The patient complained of constant pain in the left side. There was no retention and no excess of urine, and he at no time voided blood with the urine. The symptoms in the main were negative. The amount of pus was not enough to indicate a cyst of the kidney or any great amount of trouble with the bladder. He had no elevation of temperature; there was no cardiac disease, but an anæmic condition developed. His appetite and digestion failed, there was a gradual loss of flesh, and he wasted away and died.

The autopsy revealed a tubercular deposit in the upper lobe of the left lung with two or three small recent cavities, but no other disease except the lesion of the left kidney, ureter and bladder, which was presented for examination. The left kidney is greatly enlarged and contains small cystic cavities. The ureter is pervious, although the passage is small. It does not communicate with the bladder. The kidney is enormously enlarged and lobulated. The bladder is thickened somewhat, and at the point where the left ureter ought to enter there is a hard, fibrous mass, which would very easily occlude the ureter.

Professor A. E. Hoadley opened the discussion. He said: My opinion, after looking at the specimen under the microscope, is that it is unquestionably a case of tuberculosis of the kidney, primarily with extension down through the ureter into the bladder.

We have two varieties of tuberculosis of the kidney, one a *general parenchymatous tuberculosis*, the other denominated *scrofulous pyelitis* or *pyelonephritis*, which is also a tubercular condition. In the former the tubercular points are disseminated through the structure of the kidney and appear as minute miliary nodules that can be seen by the naked eye. These are masses of tubercular granulation tissue situated upon the terminal filaments of the arterioles; they infiltrate the vascular network and the connective tissue, compressing the tubuli and thus interfering with the function of the kidney. Such a condition is most common in males, and occurs usually before the age of puberty. It is found, however, at all ages. I believe Professor Danforth's case comes under the head of the "scrofulous variety." This form of the disease makes its appearance in the submucous connective tissue of the apices of the papillæ of the kidney, from there extending into its structure, forming cheesy, putty-like mases. It is a question with pathologists whether these masses are an aggregation of miliary tubercle and subsequent degeneration of them, or whether it is a retrograde change in the products of inflammatory action. Some pathologists believe both conditions may exist, inflammatory at first, then tubercular, with subsequent destructive changes. These cheesy masses increase in size and number, involve the entire structure of the kidney from the pelvis or calices out towards the cortical substances. Ultimately they break down and discharge a cheesy-like material into the cavity of the pelvis; the mucous membrane being swollen, this putty-like material plugs up the ureter, and the whole kidney may become one large abscess cavitiy, or may be filled up with numerous abscess cavities, as is the case in this specimen. The caseous degeneration passes out through the structure of the kidney, the organ becomes enlarged and lobulated and a section through one of the lobules shows a pyramidal mass of putty-like material. These small abscess cavities which result will wash out readily and leave a ragged surface in which there is no resemblance of kidney structure. The condition of this pelvis is what is denominated by the older pathologists as scrofulous ulceration, and is more apt to be confined to one kidney; while the general parenchymatous miliary infiltration of the tubercular material is an evidence of constitutional disease which may invade one or perhaps many other organs.

The more localized the disease the more it is within the domain of surgery. Where there is evidence of interference with the function of the kidney as a result of tubercular infiltration, it is useless to attempt to do anything in a surgical way. The disease is too general. Where the disease is confined to the pelvis of the kidney and produces a large tubercular abscess, opening the abscess and draining it is a good practice. But nephrectomy, as a rule, is not justifiable. It has been demonstrated that where the kidneys are involved a tubercular diathesis exists; that it is only a question of time when other organs will become implicated.

When I first examined the hard, well-defined mass in the bladder at the orifice of the ureter, I thought that it was a carcinoma with extension up along the ureter, but when we know it is a tubercular condition, we know that the extension takes place in the other direction. If it commences as a general tubercular condition, it rapidly extends and involves the ureter, bladder, prostate and vesiculæ seminales. The scrofulous ulceration of the pelvis of the kidney is not apt to extend to the ureter. It will destroy the kidney before it will involve the ureter any further than it does by plugging it up.

Here we have both types represented—a general parenchymatous miliary tubercular infiltration, and an ulcerative condition of the ureter, and a large aggregation of tubercles in the bladder as a result of extension of the pathological process.

We sometimes find in the urine, along with this putty-like material, pus and blood, and some *débris* of the kidney, but never casts of the tubuli in tuberculosis of the kidney. If we find albumen, the urine is heavy, cloudy, contains a good deal of deposit, and is not clear as in Bright's disease. If we can find casts of the tubuli of the kidney, we can almost, with unerring certainty, diagnose against tuberculosis. All the other symptoms, such as pain, strangury, etc., are so variable that they are of little practical value as a means of diagnosis. There are no positive indicative symptoms of the presence of the tuberculosis of kidney.

Dr. J. M. Patton: The main question of interest in the pathology is whether this specimen is one of tuberculosis, or whether it is a lymphadenoma. Tuberculosis of the kidney is rare. It is said by most authors never to occur primarily, and is generally considered to be secondary to disease of the lungs, the testicle, or of the prostate gland. The involvement of the lung in this case is very interesting. Tuberculosis of the lungs, as a rule, invades a much greater area of lung tissue than is involved in this case. The infiltration is more diffuse. The cavities may not be large, but they are usually numerous. It would take but a very short time for a piece of lung three or four inches square to become infiltrated with tuberculous deposit—probably not over three to five months from the time it started in the lung—and in case the patient should live a year subsequent to the active onset of the disease, I should expect a much greater portion of lung implicated than is apparent in Professor Danforth's case. As there were no sections of the lung made, we have no positive evidence that the disease was tuberculosis. Tuberculosis of the kidney, beginning, as Professor Hoadley has said, in the medullary portion, is very rapid in its growth, and, as a rule, cavities are found early. In this specimen there is a small rim of cortical substance that is in a comparatively healthy condition. That, of itself, would show that the disease has not so thoroughly infiltrated the part as is customary in tuberculosis of the kidney.

The section taken from the bladder shows an increase in the connective and muscular tissues—a mixture of adenoma, myoma and connective tissue. It is much firmer than that in the kidney, and there is a difference in its structure. The section taken from the kidney shows chiefly broken-down granulation tissue.

In order to make a diagnosis of tuberculosis by the microscope, it is necessary to have the tuberculous cells. Whether that was an unfortunate section or not, I do not know. There is no cell, large or small, that shows distinct tubercular tissue—no branching cells surrounded by the lymph—filled stroma peculiar to tuberculosis.

As far as it is possible to determine upon these sections, without a more thorough and careful microscopical examination, I should say it was a lymphadenoma, and that the growth in the bladder was secondary to that of the kidney, and is a mixture of adenoma and myoma. I believe the growth in the bladder to be secondary, for if it had been a primary growth there would have been dilatation of the ureter and pelvis of the kidney and pressure atrophy of its medullary portion.

### TYPHLITIS.

Dr. W. T. Thackeray reported a case of typhlitis. A. S. D., age 23, book-keeper; father died of tuberculosis in 1868; mother died of cancer in 1881; patient emaciated, anæmic; was attacked in January, 1888, in the city of Minneapolis, with what was said to be *winter cholera*. Present attack commenced March 21st with intense pain in the right iliac fossa. This was thought to be due to constipation, and patient took two of Carter's little liver pills; as pain continued after action, he took 15 drops tr. opium. I was called on March 22d, and diagnosed *typhlitis*. March 22d, 4 p. m., patient complained of an intense pain in the right iliac fossa, extending down to within one inch of Poupart's ligament, upwards one inch above crest of ilium, the tenderness covering a space of about 3x5 inches; temperature 100° 3.5; urine normal; patient had passed a very restless night. Treatment (No. 1): aconitine 1–500; veratrine 1–134. (No. 2) strychnine arsen. 1–134 to be given alternately every hour.

March 23d, 9 a. m., discontinued No. 1—pulse 83; temperature 98°. A surgical consultation was suggested, and Dr. W. T. Belfield called. An examination per rectum was made, but no serious swelling found; elevation of temperature was thought to be due to excitement of examination, as temperature arose to 100° 4.5. March 24, area of tenderness decreased; discontinued

No. I and added to No 2 qui. hydrofer 1-67, to be taken every hour. Temperature was 98° 2.5. The high temperature at 5 p. m of 23d was controlled at 11 p. m of same date; gave codeine 1-67 occasionally to relieve coughing, which habitually annoyed patient. March 24th, continued No 2; temperature 99° 1.5; 25th, area of tenderness decreased; temperature 98° 4.5; several callers during the day had caused some slight excitement. 26th, continued No. 2; temperature 99° 1.5, the elevation being due to the excitement of the previous day. 27th, area of tenderness 1 x 1½ inches; hawthorne water; No. 2 continued; temperature 98°. 27th, p. m., continued No. 2 every two hours, and added iodoform 1-6; temperature 98° 3.5; had an action of the bowels and in the fæces was found a bean, the shell of which was very hard; the patient had eaten baked beans on the 18th of March. 28th, 9 a. m., area of tenderness imperceptible by palpation or pressure; temperature 98° 2.5; patient allowed to sit up in a chair. April 1st, went out on the street perfectly recovered.

